# Formula diet driven microbiota shifts tryptophan metabolism from serotonin to tryptamine in neonatal porcine colon﻿

**DOI:** 10.1186/s40168-017-0297-z

**Published:** 2017-07-14

**Authors:** Manish Kumar Saraf, Brian D. Piccolo, Anne K. Bowlin, Kelly E. Mercer, Tanya LeRoith, Sree V. Chintapalli, Kartik Shankar, Thomas M. Badger, Laxmi Yeruva

**Affiliations:** 1 Arkansas Children’s Nutrition Center, 15 Children’s Way, Little Rock, AR 72202 USA; 2 Department of Pediatrics, University of Arkansas for Medical Sciences, Little Rock, USA; 3 Arkansas Children’s Research Institute, Little Rock, USA; 4 Department of Biomedical Sciences and Pathobiology, Virginia-Maryland Regional College of Veterinary Medicine, Blacksburg, USA

**Keywords:** Formula, Piglets, Immune response, Serotonin, Tryptamine, Colon, Microbiota

## Abstract

**Background:**

The gut microbiota of breast-fed and formula-fed infants differ significantly, as do the risks for allergies, gut dysfunction, and upper respiratory tract infections. The connections between breast milk, various formulas, and the profiles of gut bacteria to these childhood illnesses, as well as the mechanisms underlying the effects, are not well understood.

**Methods:**

We investigated distal colon microbiota by 16S RNA amplicon sequencing, morphology by histomorphometry, immune response by cytokine expression, and tryptophan metabolism in a pig model in which piglets were sow-fed, or fed soy or dairy milk-based formula from postnatal day (PND) 2 to 21.

**Results:**

Formula feeding significantly (*p* < 0.05) altered the colon microbiota relative to the sow feeding. A significant reduction in microbial diversity was noted with formula groups in comparison to sow-fed. *Streptococcus, Blautia, Citrobacter, Butrycimonas, Parabacteroides, Lactococcus* genera were increased with formula feeding relative to sow feeding. In addition, relative to sow feeding, *Anaerotruncus, Akkermansia, Enterococcus, Acinetobacter, Christensenella,* and *Holdemania* were increased in milk-fed piglets, and *Biliophila, Ruminococcus, Clostridium* were increased in soy-fed piglets. No significant gut morphological changes were noted. However, higher cytokine mRNA expression (BMP4, CCL11, CCL21) was observed in the distal colon of formula groups. Formula feeding reduced enterochromaffin cell number and serotonin, but increased tryptamine levels relative to sow feeding.

**Conclusion:**

Our data confirm that formula diet alters the colon microbiota and appears to shift tryptophan metabolism from serotonin to tryptamine, which may lead to greater histamine levels and risk of allergies in infants.

**Electronic supplementary material:**

The online version of this article (doi:10.1186/s40168-017-0297-z) contains supplementary material, which is available to authorized users.

## Background

Breastfeeding is associated with a variety of positive health outcomes in children, such as: lower incidence of diarrhea; influenza; ear infections; and respiratory tract infections [1]. It has been suggested that breast-fed infants have advanced immune system development compared to formula-fed infants [2, 3]. The microbiota acquired in early life has been reported to be important for mucosal immune response and tolerance, suggesting linkage to mucosal inflammation, autoimmunity, and allergy disorders [2, 3]. However, the underlying mechanisms remain to be fully elucidated. Complementary to human infant studies, formula-fed piglets compared to sow-fed piglets differed significantly in: diarrhea frequency; levels of mucosal IgG and IL-2; lactic acid bacteria/E.coli ratio [4]; and counts of colonic *Clostridium cluster IV and Bacteroides vulgatus* [5], all of which are consisent with microbiota differences found between human infants fed breast milk or formula. Comparison of infants’ microbiota with piglet microbiota indicates that predominant phyla observed in breast-fed and formula-fed infants are *Bacteroides* and *Fimicutes,* which are also the abundant phyla in sow-fed and formula-fed piglets. However, *Actinobacteria* is also predominantly observed in breast-fed and formula-fed infants but very small percent was observed in sow-fed piglets and none in formula-fed piglets [6]. Furthermore, previous report on metagenome profile comparison between porcine fecal and human fecal samples showed 70% functional similarity [7]. Given the similarity of the infant and piglet intestine [8], the neonatal piglet is arguably the most useful model to explore the interaction between infant diets and gut microbiota to understand the mechanisms underlying health complications in infants.

Although diet-associated alterations in the composition of the infant gut microbiota are well documented, the functional effect on gastrointestinal tract physiology and immune response remains to be fully elucidated. One potential mechanism is an interaction of the microbiota with the host gastrointestinal tract and beyond through “xeno-metabolites” produced through microbial metabolism. For example, lower levels of SCFAs (short-chain fatty acids) were correlated with *Bacteroides, Prevotella, Clostridium clusters XIVa and IV, Bifidobacterium spp*. and *Lactobacillus* from colons of formula fed piglets [9]. Interestingly, Yano et al. reported that gut microbiota regulate host sertotonin biosythesis [10]. Several exogenous factors including specific dietary components and microbiota alter serotonin release [10, 11]. However, it is not currently known whether the serotonergic pathway is altered by the microbiota in infants and, thus, may be a potential pathway influencing gut immune system development and function.

Previously, we demonstrated that neonatal pigs fed formula differed from sow-fed piglets in small intestine morphology and had decreased lymphoid follicle size and germinal centers in Peyer’s patch and ileum tissue [12]. Herein, we explore the effects of formula on the colon microbiota and the impact on colon morphology and immune response, as well as metabolites involved in the serotonin pathway and serotonin metabolism in the colon.

## Methods

### Animal housing and feeding

Thirty-six Yorkshire/Duroc crossbreed piglets (2 days old, *n* = 6/group/gender) were used in this study. The animals were maintained by the ethical guidelines established and approved by Institutional Animal Care and Use Committee (IACUC) of University of Arkansas Medical Sciences. The Animal feeding and diet composition details and body weights are published in our previous paper [12]. In brief, piglets were randomized into three groups, i.e., group 1-sow fed; group 2-soy-based formula (soy) (Enfamil Prosobee Lipil powder; Mead Johnson Nutritionals, Evansville, IN) fed; group 3-cow’s milk-based formula (milk) fed (Similac Advance powder; Ross Products, Abbott Laboratories, Columbus, OH). Piglets were fed formula milk (1.047 MJ/kg/day) from small bowls on a fixed schedule (i.e., 1st week every 2 h, 2nd week every 4 h, and 3rd week every 6 h) until sacrifice on a postnatal day (PND) 21. These piglets were anesthetized with isoflurane, blood was collected and then sacrificed by exsanguination. All tissues and contents were flash frozen in liquid nitrogen and stored at −80 °C until use, while a portion of tissues was fixed in formalin for histomorphometric analyses.

### Microbiota analysis using 16sRNA amplicon sequencing

Distal colon contents were subjected to DNA isolation using Qiagen DNA isolation kit [13]. Amplicons were generated by PCR of variable region 4 (V4) of bacterial 16S rRNA genes. Multiplex sequencing was carried out with an illumina platform. QIIME software was used for clustering of V4 rRNA reads at 97% nucleotide sequence. PiCRUST (phylogenetic investigation of communities by reconstruction of unobserved states) was used to generate a profile of putative functions (via metagenome prediction) from the 16srRNA OTU data [14]. OTUs were picked from a demultiplexed fasta file containing the sequences for all distal colon contents samples using the closed-reference protocol where we searched sequences against the GG reference OTUs at 97% percent identity. These OTUs were normalized by the predicted 16S copy number. BIOM table containing the predicted metagenome for each sample was attained. We used STAMP (statistical analysis of metagenomics profiles) [15, 16] software to determine statistical significance of functional metagenomics prediction data [15, 16]. The significant differences in functional category relative abundances among sow, soy, and milk formula fed samples at level-3 profile were determined by ANOVA followed by Tukey-Kramer post-hoc test with effect size Eta-squared to test.

### Colon morphology and immune response

Colon samples were embedded in paraffin after dehydration. Six-micrometer-thick sections were stained with hematoxylin and eosin and used for histomorphometric analysis. The digital images were captured using Aperio microscope scanner (Aperio CS) and were used to measure the crypt height, crypt density (crypt number per mm length of tissue), full thickness of membrane, thickness of granular muscularis, and colon circumference in the sections of distal colon using ImageScope (Version 12.1.0.5029, Aperio Technologies) and Image J (Image J 150b, NIH, USA). Gene expression was measured by real time PCR (Additional file 1: Table S2).

### Western Blot

Frozen colon tissue (~100 mg) was homogenized in cell lysis buffer (500 μl) (Cat EPX-99999-000 eBioscience, San Diego, USA) containing 0.1% proteinase inhibitor cocktail (Sigma, St. Louis, MO) and 1% NP40, in ceramic bead tubes using Fast Prep-24^TM^ 5G machine (M.P. Biomedical LLC, California, USA) at a speed 6.0 ms for 30 s (twice). Samples were centrifuged at 12,000 g for 15 min at 4 °C, supernatants were collected and protein concentration was determined using Bio-Rad protein estimation kit (BioRad). 100 μg of protein was subjected to Western Blot analyses using 8% acrylamide gel. Membranes were probed with anti-pig primary antibodies raised in rabbit overnight at 4 °C and subsequently incubated with 1:10,000 dilution of goat-anti-rabbit HRP for 1 h at room temperature (BioRad Laboratories Inc., California). We used 1:1000 dilution of anti-VE-cadherin, anti-catenin, anti-HSP 27 (Abcam, Cambridge, MA) as primary antibodies. Detection was performed using a chemiluminescence system (super signal west chemiluminescent substrate, Thermo Scientific). Image Quant software (Image Quant TL 8.1 Version) was used for densitometric analysis. Anti-rabbit vinculin (Abcam ab73412) that cross reacts with pigs was used as a housekeeping protein for normalization of a target protein.

### Intestinal alkaline phosphatases activity measurement

The supernatants of distal colon contents, duodenum contents, and serum were used to measure intestinal alkaline phosphatases (ALP) using commercial kit (Anaspec CA, AS72146). This kit uses p-nitrophenyl phosphate (pNPP) as a phosphatase substrate which turns yellow (λmax = 405 nm) when dephosphorylated by ALP. In brief, 40 μl of sample, 10 μl of assay buffer and 50 μl of 5mM pNPP solution were mixed and incubated in the dark at room temperature for 30 to 60 min. The reactions were stopped by adding 50 μl Stop Solution and plates were read at 405 nm. The serial diluted pNP (5μ Mol) and pNPP (mM), ALP enzymes (2ng) were used for preparation of substrate, product and enzyme standard curves respectively. Absorbance readings were taken immediately and every 5 min for 60 min to obtain enzyme kinetics. Alkaline phosphatase activity in the test samples was calculated as enzyme activity (moles substrate/min) = (V × vol)/(e x l). V is reaction velocity (OD 405/min), vol is reaction volume in liters, e is the extinction coefficient of pNPP, 1.78 × 10^4^ M^−1^cm^−1^and l is the path length of light through the sample in cm (for 100 ml sample, *l* = 0.5 cm). This equation determines enzyme activity in terms of moles of substrate conversion per minute.

### Immunohistochemistry and Immunofluorescence

To see the expression of HSP27, immunohistochemistry were carried out with 6 μm thick sections of distal colon. Anti-rabbit HSP27 (1:1000 dilution), ABC kit, DAB kit, and Texas Red labeled - anti-rabbit IgG (Vector Laboratories, Inc, Burlingame, CA) were used to perform immunohistochemistry. Olympus microscope (BX50) with 3CCD color camera (DCX-976MD) were used to capture the images. To see the expression of 5HT producing enterochromaffin cells in distal colon, 6 μm thick sections of distal colon were deparaffinized and rehydrated. Citra Plus or Dako Target Retrieval (pH6) solution was used for antigen retrieval. After blocking for 1hour (hr) with 5% donkey serum in PBS, tissue sections were incubated for 3hr at room temp with a cocktail of 1:2000 rabbit anti-5-HT antibody (ImmunoStar, 20080) and 1:250 mouse anti-chromogranin A (Abcam, ab199014), prepared in PBS with 5% donkey serum. Tissue sections were then incubated with secondary cocktail containing 1:100 donkey anti-rabbit Alexa 488 (Jackson Immuno, 711-545-152) and 1:100 donkey anti-mouse Alexa 594 (Jackson Immuno, 715-585-150) in 1x PBS for 1 h at room temp. The sections were mounted with prolong gold containing DAPI. The images were acquired using fluorescence microscope (Olympus BX51TRF). The green and red fluorescent cells indicated 5-HT and CgA expressing cells, respectively. We counted the number of 5-HT expressing EC cells (5HT+/CgA+) (yellow color) and a total number of EC cells (CgA+) cells.

### Serotonin ELISA

Frozen distal colon tissues (~80 mg/400 ul) or distal colon contents (~50 mg/300 ul) or duodenum contents (~50 mg/300 ul) were homogenized in ceramic bead tubes containing cell lysis buffer (Cat EPX-99999-000 eBioscience, San Diego, USA) with 0.1% proteinase inhibitor cocktail (Sigma, St. Louis, MO), 1% NP40 and 0.1% ascorbic acid using Fast Prep-24^TM^ 5G machine (M.P. Biomedical LLC, California, USA) at speed 6.0 ms for 30 s (twice). Samples were centrifuged at 12,000 g for 15 min at 4 °C, supernatants were collected, and total protein concentration was determined using Bio-Rad protein estimation kit (BioRad). Serotonin levels were detected in clear supernatant of distal colon tissues, distal colon contents, duodenum contents, diet samples, serum and urine by ELISA according to the manufacturer instructions (Eagle Biosciences SEU39-K01). The plate was read at 450 nm (reference wavelength 600 nm) using a microplate spectrophotometer (Benchmark plus, BioRad). The data were normalized with total protein and tissue weight.

### Serotonin metabolites measurements

The presence and relative abundance of serotonin metabolites in serum, urine, and colon contents of neonatal piglets was determined using an ultra-high-performance liquid chromatography (UHPLC) system coupled to a high-resolution accurate mass spectrometer (HRAM). All instrumentation, columns, and software used are products of Thermo Fisher Scientific (Waltham, MA). All solvents used were of optimal grade and purchased from Fisher Scientific (Pittsburgh, PA). Analytical HPLC grade compounds were obtained from Sigma-Aldrich (St. Louis, MO).

Metabolites in serum (100 μl) were extracted in methanol (2:1); colon contents (~50 mg) were homogenized in 50% aqueous methanol and extracted in acetonitrile (2:1). All extracts were dried under a nitrogen stream and reconstituted in 5% aqueous methanol spiked with an internal standard (Lorazepam, 500 ng/ml). Urine samples were first analyzed for creatinine using a commercially available assay (#STA-378, Cell Biolabs, San Diego, CA). Urines were then normalized to the lowest creatinine concentration (0.036 mg/ml) by dilution in water before analysis.

For serum and colon content metabolites, chromatographic separation was performed on an Ultimate 3000 UHPLC system fitted with a Hypersil GOLD C18 reversed-phase column (100 × 2.1 mm, 1.9 μ). Mobile phases consisted of 0.1% formic acid in water (A) and 0.1% formic acid in acetonitrile (B). The flow rate was set at 0.4 ml/min with an elution gradient as follows: 0 to 1% B from 0.0 to 2.0 min; 1 to 20% B from 2.0 to 6.5 min; 20–95% B from 6.5 to 11.5 min; 95 to 99% B from 11.5 to 13.5 min; 99–1% B from 13.5–16.5 min; hold at 1% B until 20.0 min. Sample injection volume was 5 μl. Urine metabolites were separated using a Hypersil GOLD (50 × 2.1 mm, 1.9 μ) column using the following elution gradient: 0 to 20% B from 0.0 to 4.0 min; 20–100% B from 4.0 to 9.0 min; 100 to 0% B from 9.0 to 12.0 min. Sample injection volume was 5 μl.

Detection was carried out on a Q-Exactive Hybrid Quadrupole-Orbitrap mass spectrometer with data acquisition executed using Xcalibur 4.0 software. All samples were analyzed by positive electrospray ionization (ESI+) Full-MS scan mode. Nitrogen as sheath, auxiliary, and sweep gas was set at 50, 13, and 3 units, respectively. Other conditions included: resolution, 70,000 FWHM; AGC target, 3e6 ions; maximum injection time, 200 ms; scan range, 50–750 m/z; spray voltage, 3.50 kV; and capillary temperature, 320 °C. All data was then processed using TraceFinder 3.3 software. Raw files were imported and screened against a compound database generated from ESI+ Full-MS scans of standard mixtures of 5-hydroxytryptophan (5HTP), 5-hydroxyindoleacetic acid (5HIAA) and tryptophan. Metabolites were identified by exact mass (±3 ppm) and a retention time (±15 s) as shown in Additional file 1: Table A. Following identification, all samples within each biological matrix were pooled together. These pooled samples were then analyzed by ESI+ Full-MS with PRM (parallel reaction monitoring) targeting the five compounds of interest. MS2 spectra was generated at three fixed collision energies-10, 30, and 45%. Raw files were imported and individual MS2 spectra were matched to an in-house MS2 database created in Library Manager 2.0 with a match score >80% to the compound of interest (Additional file 1: Table S6).

### Tryptamine and its metabolite measurements

Tryptamine levels were evaluated as previously described by Sangwan et al. (1998) [17, 18]. A homogenate of distal colon content or duodenum contents (~50 mg) was prepared in assay buffer containing 0.1 M sodium phosphate (pH 7.5), 5 mM beta-mercaptoethanol, 5 mM thiourea and 1 mM ethylenediaminetetraacetic acid followed by centrifugation for 30 min at 10,000 g. The clear supernatant of distal colon contents (25 μl) or duodenum contents (25μl) or serum (75 μl) or urine (75 μl) or diet samples were diluted up to 250 μl with assay buffer and used for tryptamine analyses. Standard curve was generated by serial dilution of 1 mg/ml tryptamine (Sigma 193747) in assay buffer. The fluorescence intensity of samples was determined using a fluorescence spectrometer (Molecular Device Spectra Max Gemini XPS) with excitation and emission wavelengths at 280 and 360 nm, respectively.

Indole acetic acid (IAA) concentration was measured by using Salkowski method [19, 20]. 20 μl of the supernatant was diluted with 80 μl of phosphate buffer and 440 μl of reagent R1 was added. Reagent R1 (Salkowski reagent) contains 12 g of FeCl_3_ per liter of 7.9 M H_2_SO_4_. Red color formation was quantified as the absorbance at a wavelength of 540 nm in a microplate spectrophotometer (Benchmark plus, BioRad). Standard curve was generated by serial dilutions of a 5mM IAA stock solution in phosphate buffer.

### Statistical Analysis

Microbiota OTU reads were imported into R version 3.2.1 and all statistical analysis were performed using the vegan and phyloseq packages unless specifically noted. OTU richness was measured by Chao1 and OTU diversity was measured by several diversity indices (Shannon, Simpson, Inverse Simpson, and Fisher). Group differences in α-diversity (richness and diversity) were assessed by ANOVA using the stats package. Between-specimen diversity (β-diversity) was assessed by calculating a matrix of dissimilarities using the Bray-Curtis method and then visualized using non-metric multidimensional scaling (NMDS). Group differences in β-diversity was assessed using permutational multivariate analysis of variance (PERMANOVA) with 999 permutations. Group differences among genus level OTUs were assessed by pairwise comparisons on read counts using Negative Binomial Wald Tests from the DESeq2 package. OTU relative abundance is given as median % relative abundance when described in text. All statistical tests used on 16S-rRNA gene sequencing data were considered significant at *α* ≤ 0.05. All tests were corrected for multiple comparisons using the false discovery rate (FDR) correction by Benjamini and Hochberg. Histomorphometry, protein expression, serotonin, and tryptamine measurements were analyzed by one/two way ANOVA followed by post hoc test (Tukey’s) using GraphPad software. Data are presented as mean ± SEM and are considered significant at *p* < 0.05. *n* = 12/group until/unless indicated. Associations among selected variables were assessed with Spearman’s correlations.

## Results

### Formula alters microbiota diversity and relative abundance in piglet colon

**Fig. 1:**
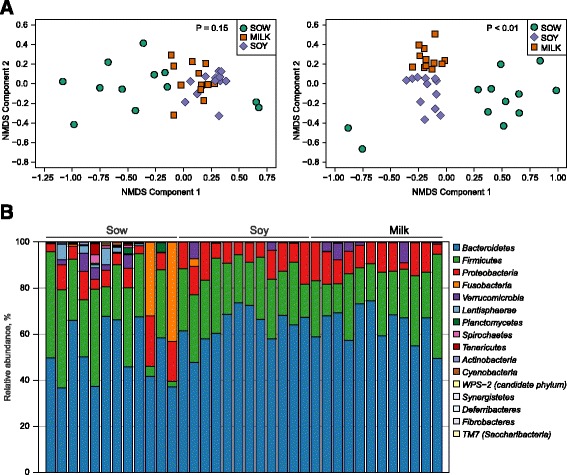
Formula diets alter the colon microbiota relative to sow diet in porcine neonates. **a** NMDA plot of microbial diversity displays the separation of sow, soy, and milk from each other at the phylum and the genus level. **b** *Stacked bar chart* shows sow, soy, and milk groups microbial relative abundance in the distal colon at the phylum level

**Fig. 2:**
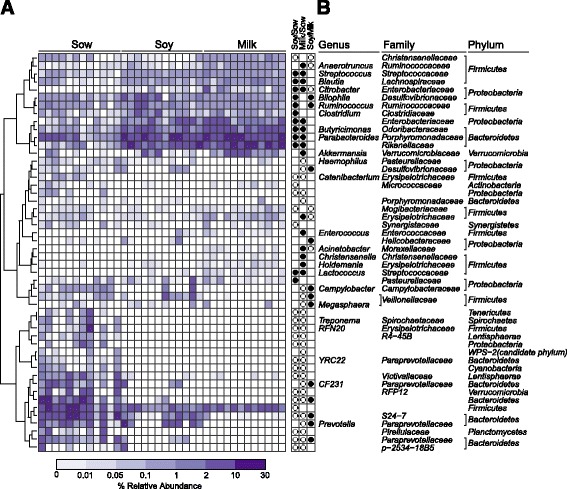
**a** Heat-map displays relative abundances of the genus that had a significant diet effect. Genera are ordered by hierarchical clustering of Bray-Curtis dissimilarities. Significant differences are expressed as *circles* in panel **b**. *Closed black circles* represent genus that were greater in the column numerator relative to the denominator. *White circles* represent genus that were lower in the column numerator relative to the denominator (*p < 0.05)

Sample richness as evidenced by Chao1 index was significantly lower in formula groups’ at all taxonomical levels except at the order level. Sample diversity (Shannon index) inferred significance only at the phylum level (Additional file 1: Table S1). Non-metric multidimensional scaling (NMDS) plots of β-diversity revealed no dietary difference at the phylum level; however, a significant diet effect was observed at the genus level with sow-fed animals discriminated from soy- or milk-fed piglets (Fig. 1a). At the phylum level a total of 16 phyla were found in sow-fed, but not in soy- or milk-fed piglets, (Fig. 1b) suggesting a reduction of microbial diversity in formula-fed piglets. *Bacteroidetes* represented the most abundant phyla in all groups (52.9, 65.1, and 66.1% in sow-, soy- or milk-fed piglets, respectively), followed by *Firmicutes* (23.9, 22.6, 20.5%) and then *Proteobacteria* (9.3, 10.9, 10.8%), respectively (Fig. 1b). Detailed description of relative abundances for class, order, and family levels are discussed in supplemental results (Additional file 1: Figure S1A-1C). At the genus level, 50 total OTUs were differentially impacted by diet (Fig. 2). Relative to sow feeding, 40 OTUs were significantly altered with milk-formula feeding and 33 with soy formula feeding. Of these, 24 OTUs were differentially altered in both formula groups compared to sow-fed piglets. In addition, 18 OTUs were altered significantly in milk-fed piglets relative to soy-fed. *Streptococcus, Blautia, Citrobacter, Butrycimonas, Parabacteroides, Lactococcus* were all identified genera that were increased with soy or milk formula feeding relative to sow feeding. *Butrycimonas* and *Parabacteroides* were greater in formula-fed animals by at least a factor of five (*Butrycimonas:* 0.09, 1.95, and 3.63% in sow, soy, and milk, respectively; *Parabacteroides* 1.57, 8.52, 8.47% in sow, soy, and milk, respectively). In addition, relative to sow-fed piglets, *Biliophila, Ruminococcus, Clostridium* were greater with soy formula feeding while *Anaerotruncus, Akkermansia, Enterococcus, Acinetobacter, Christensenella, Holdemania* were greater with milk feeding. Almost half of significant OTUs were decreased in formula-fed piglets relative to those who were sow-fed (Fig. 2; 20 OTUs). Twelve genera, including *Treponema*, *Catenibacterium*, *RFN20*, *YRC22*, and 8 unassigned OTUs, were present only in sow-fed piglets (i.e., completely absent in soy and milk-fed piglets (Fig. 2). Altogether, changes in microbial composition and diversity indicate that neonatal diet shapes the colon microbiota.

### Formula impact on colon morphology and immune response piglets

**Fig. 3:**
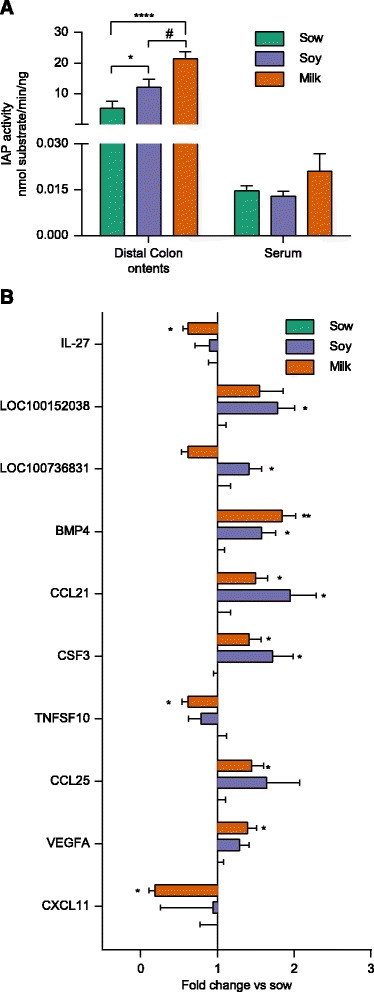
Formula diets significantly alters tissue immune response relative to sow diet in porcine neonates. **a** *Bar graph* shows intestine alkaline phosphatase activity in distal colon (DC) contents and serum (analyzed by two-way ANOVA followed by tukey’s post hoc test). **b** *Bar graph* shows the expression of different cytokines and chemokines in distal colon of sow-, soy-, and milk-fed piglets (analyzed by multiple *t* test). (*p < 0.05, **** *p*<0.0001  for formula diet in comparison to sow fed, ^#^ *p* <0.05 for milk diet in comparison to soy diet)

No differences were observed in gross colon-morphology among the diet groups (Additional file 1: Figure S2A). A significant increase in colon length (Additional file 1: Figure S1B) and circumference (Additional file 1: Figure S1E) and a decrease in crypt depth/tissue thickness ratio (Additional file 1: Figure S1C) were observed in soy-fed relative to sow-fed groups. No significant differences were observed in colon crypt counts (Additional file 1: Figure S2D). To determine the membrane integrity, several membrane proteins e.g. VE-Cadherin, β-catenin, HSP-27, and alkaline phosphatase were measured by western-blot and cytokine gene expression was measured by real time PCR. No significant difference in expression of cadherin proteins was observed among the groups (data not shown). Interestingly, HSP-27 and β-catenin showed a trend for an increased protein expression in soy relative to sow-fed piglets (Additional file 1: Figure S2F-H). We confirmed HSP-27 expression by Immuno-staining (Additional file 1: Figure S2H). We observed increased alkaline phosphatase activity in distal colon contents especially in milk group (Fig. 3a) with no difference in serum. Furthermore, in soy or milk group upregulation of BMP4, CCL21, CSF3, CCL25, and VEGFA and down regulation of TNFSF10 and CXCL11 was observed relative to sow-fed group, suggesting that formula diet impacts the immune system (Fig. 3b, Additional file 1: Table S2).

### Predictive functional profiling of neonatal diet driven microbiota

**Fig. 4:**
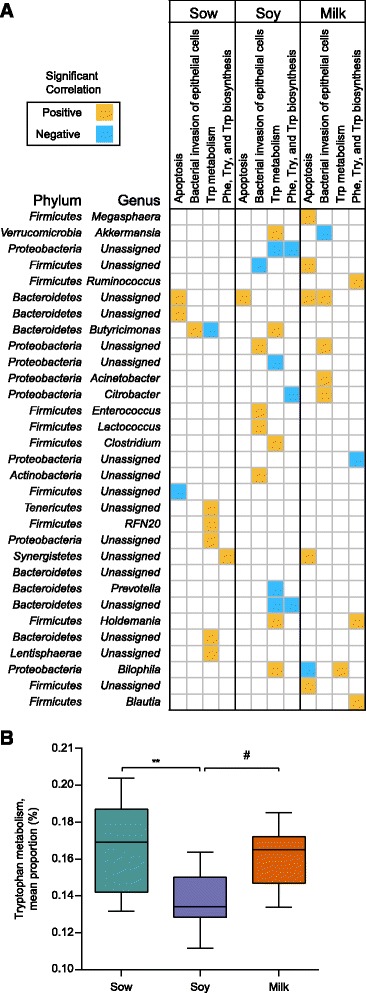
Correlation matrix of microbiota to tryptophan and cellular processes. **a** Significant correlations among genera and tryptophan metabolism, apoptosis, and bacterial invasion of epithelial cells are shown. The significant Spearman correlation coefficients are displayed as *ellipses. Let* and *right directions* of ellipses indicate positive and negative correlations, respectively. **b** The *bar graph* shows the prediction analyses of tryptophan metabolism. (**p < 0.05 for milk diet in comparison to sow-fed, ^#^ *p* < 0.05 for milk diet in comparison to soy diet)

As microbiota shift did not appear to impact the colon morphology or colon membrane protein expression, we assessed the functional significance of the microbiota using PICRUSt. The analyses predicted the functional composition of a metagenome using marker gene data and a database of reference genomes. A significant difference was observed in 43 pathways among the diet groups based on cellular process, environmental, genetic information processing, metabolism, and human diseases (Additional file 1: Figure S3). Our analyses predicted significant higher apoptosis, and bacterial invasion of epithelial cells in soy- or milk-fed groups in comparison to the sow-fed among the cellular and environmental pathways predicted (Additional file 1: Figure S3, level 1). Correlation analyses revealed a significant positive association of apoptosis with *Bacteroidetes* across diets (Fig. 4a). Moreover, the milk-fed group demonstrated a positive correlation to apoptosis with *Megasphaera,* and unassigned genus from *Firmicutes* and Synergistetes phyla*.* In the sow-fed group *Butyricimonas* was positively correlated with bacterial invasion of epithelial cells while in the soy fed *Enterococcus, Lactococcus* genera and unassigned genera from *Proteobacteria* and *Actinobacteria* phyla were positively correlated. In the milk group, *Acinetobacter, Citrobacter* genera and unassigned genera from *Bacteroidetes* and *Proteobacteria* phyla were positively correlated with bacterial invasion of epithelial cells.

### Impact of diet on tryptophan metabolism

Correlation analyses (Fig. 4a) indicates that *Akkermansia, Butyricimonas, Clostridium, Holdemania, and Bilophila* were positively correlated with tryptophan metabolism in the soy-fed, while only *Bilophila* genus correlated with tryptophan metabolism in the milk group. However, unassigned genus from phyla *Tenericutes, Proteobacteria, Bacteroides,* and *Lentisphaerae* in addition to *RFN20* genus from *Firmicutes* phylum were positively correlated with tryptophan metabolism in the sow-fed group but not in formula-fed groups. In addition, *Butyricimonas* was negatively correlated with tryptophan metabolism in the sow-fed group while unassigned genus from *Proteobacteria* and *Bacteriodetes* phyla were negatively correlated with tryptophan metabolism in the soy group. Among the metabolism pathways predicted, we explored mainly the tryptophan metabolism (Additional file 1: Figure S3, Fig. 4b).

### Formula suppresses the host serotonin level in colon of piglets

**Fig. 5:**
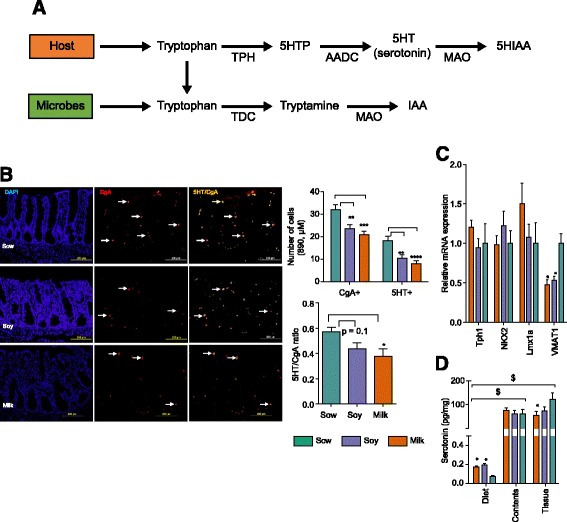
Formula diets alter the serotonin level in porcine neonates fed with soy or milk formula relative to sow diet. **a** *Cartoon* displays the tryptophan metabolism to serotonin and bacterial tryptamine and the enzymes involved in the pathway (**b**) The digital images show the immunostaining of EC cells in distal colon sections with 5-HT (*green*), CgA (*red*) and 5-HT/CgA (*yellow*). The *bar graph* displays the decrease in a number of EC cells in soy- and milk-fed groups relative to the sow-fed group. (**c**) Relative EC cell gene expression in distal colon tissue. (**d**) Serotonin level in diets (*n* = 3–4/diet group), distal colon tissue (normalized with tissue weight), distal colon contents of soy and milk groups in comparison to the sow-fed (*n* = 12/group). Data were analyzed by two-way ANOVA followed by post hoc test (Tukey’s). (*p < 0.05, **p < 0.01, ***p < 0.001, ****p < 0.0001 for formula diet in comparison to sow-fed, ^$^ *p* < 0.005 comparison of serotonin levels in diets versus contents or tissue independent of treatment group). EC = Enterochormaffin cells, CgA = Chromogranin A

Tryptophan is a precursor for both host serotonin and bacterial tryptamine biosynthesis. In the host, enterochromaffin (EC) cells synthesize >90% of serotonin in the gastrointestinal tract [21], where tryptophan is converted into 5-Hydroxytryptophan (5-HTP) and serotonin (5-HT) by the enzymes tryptophan hydroxylase (TPH) and aromatic acid decarboxylase (AADC). Serotonin catabolizes into 5-hydroxyindole aldehyde (5-HIA) and 5-hydroxyindole acetic acid (5-HIAA) in the presence of MAO (monoamine oxidase) and aldehyde dehydrogenase [22] (Fig. 5a). To understand the impact of formula diet on serotonin synthesis, we determined EC cell and serotonin producing EC cell numbers by immunofluorescence staining. Both EC cells and 5HT- were significantly reduced in the distal colon of soy- and milk-fed piglets relative to sow, while the ratio of 5-HT/CgA reached significance only in milk-fed group in comparison to sow (Fig. 5b). To determine if EC cell number is truly changed because both EC cells and L cells stain positive for CgA [23], we measured EC cell and serotonin transporter (VMAT_1_) expression by real time PCR (Tph1, Lmx1a, NKX2). No difference was observed in EC cell gene expression (Tph1, Lmx1a, NKX2) in all diet groups, however serotonin transporter (VMAT_1_) expression was decreased in both formula groups relative to sow (Fig. 5c). These data suggest that VMAT_1_ expression is possibly dependent on the amount of serotonin available and EC cell number is not affected by neonatal diet. Interestingly, distal colon tissue serotonin concentrations measured by ELISA were decreased, but no differences were found in the distal colon content (Fig. 5d). Diet groups did not differ in serum and urine serotonin levels (Additional file 1: Figure S4A) either. To determine serotonin regulation, 5-HTP and 5HIAA levels were measured. No significant differences in 5-HTP and 5HIAA levels were observed among diet groups in distal colon contents (Additional file 1: Figure S4B-D). In addition, increased tryptophan and 5HTP levels were noted in serum and urine of soy and milk groups (Additional file 1: Figure S4B-D), suggesting a diet effect in these piglets. To determine the diet contribution to serotonin levels observed, we measured serotonin in diet samples and noted that soy and milk groups have significantly more serotonin than sow diet (Fig. 4d). However, the amount of serotonin observed in three diet groups is very minimal (0.05–0.20 pg/mg) relative to distal colon tissue and contents (50–100 pg/mg; *p* < 0.005) (Fig. 5d). Altogether these findings suggest that formula diets have impact on serotonin levels observed in colon.

### Formula promotes microbial tryptamine production

**Fig. 6:**
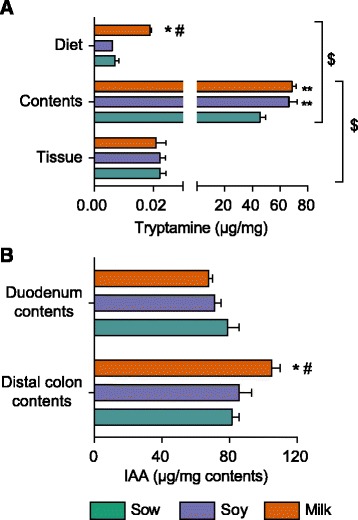
Formula diet enhances the bacterial tryptamine biosynthesis relative to sow diet in porcine neonates. **a** The *bar graph* shows tryptamine levels in diets (*n* = 3-4/diet group), distal colon contents and distal colon tissue (**b**) The *bar graph* displays indole acetic acid (IAA) in distal colon (DC) (and duodenum (DD) contents (*n*_sow_ = 7, *n*_soy_ = 9, *n*_milk_ = 11). The data were analyzed by two-way ANOVA followed by post hoc test (Tukey’s) (*p < 0.05, **p < 0.01, for formula diet in comparison to sow-fed; ^#^ *p* < 0.05, for milk diet in comparison to soy-fed, ^$^ *p* < 0.0001 comparison of tryptamine levels in diets versus contents, and in tissue versus contents independent of treatment group).

Tryptamine is produced by specific bacteria (*Clostridium, Ruminococcus*) (Fig. 5a) that utilize tryptophan. To understand if tryptophan is converted to tryptamine, distal colon contents and tissue tryptamine levels were measured. We observed signficantly more tryptamine in colon contents of formula-fed groups relative to the sow group, with no differences in distal colon tissue among diet groups (Fig. 6a). Furthermore, the level of tryptamine observed in distal colon contents (Fig. 6a) was significantly higher than in tissue in all diet groups (*p* < 0.0001 contents vs tissue). In addition, amount of tryptamine observed in the diet is significantly lower than distal colon contents independent of diet groups (*p* < 0.0001) (Fig. 6a). To determine if the small intestinal tract makes any contribution to tryptamine levels observed in distal colon contents, we measured tryptamine in duodenal contents (Additional file 1: Figure S4E). The levels of tryptamine in duodenum contents are significantly lower than in distal colon contents (*p* < 0.0001 duodenum vs distal colon) in all diet groups. In addition, tryptamine metabolizes into indole acetic acid (IAA) in the presence of MAO enzyme (Fig. 6a) [24]. Thus, we measured IAA levels in duodenum and distal colon contents, and noted significantly more IAA in distal colon contents of milk-fed group relative to the sow-fed group, but not in duodenum contents (Fig. 6b). Furthermore, in serum no significant differences were observed among groups in IAA levels, while in urine lower levels of IAA were observed in soy, and milk-fed relative to sow-fed piglets (Additional file 1: Figure S4F).

## Discussion

The gut microbiota plays an important role in health and development of infants and diet may be the most important environmental factor on gut bacterial composition. It is well known that the microbiota of breast-fed and formula-fed infants differ significantly [3]. Very little information about the relationship of diet, microbiota and immune system development and function is available for infants. We have used a pig model to study these effects. Previous reports showed that cecum from the sow-fed group was enriched with *Prevotella, Oscillibacter, and Clostridium* [21], while the formula group was enriched with *Bacteroides, Parabacteroides, and Alistipes* [25]. However, our study found that relative to sow-fed piglets, those fed with formula showed increased *Streptococcus, Blautia, Citrobacter, Butrycimonas, Parabacteroides, Lactococcus*. In addition, in milk-fed piglets, *Anaerotruncus, Akkermansia, Enterococcus, Acinetobacter, Christensenella,* and *Holdemania* were increased, while in soy-fed group *Biliophila, Ruminococcus, Clostridium* were increased relative to those who were sow-fed. Furthermore, pooled ascending colon contents from day 9 and day 17 old sow-fed or formula-fed piglets were assessed for microbiota. Data showed *Bifidobacterium* higher counts in sow-fed while, formula-fed had higher counts of *Clostridium cluster IV, XIVa* and *Bacteroides vulgatus* and our study samples are collected on day 21. The differences observed in our study to reported studies could be explained by the specific location of the contents collected, differences in housing environment, and possibly the time point samples were collected. Collectively, these data suggest that bacterial diversity is much greater in colon than in cecum, and that the microbiota profiles differ based on the location of the gut. Thus, understanding microbiota differences at each location in the gastrointestinal tract and its impact on the gut will inform the physiology of the gut in detail. Furthermore, formula feeding reduced microbial richness and diversity relative to sow feeding in the distal colon. It is possible that the higher relative abundance of genera (e.g., *Streptococcus, Blautia, Citrobacter, Butrycimonas, Parabacteroides, Lactococcus),* are known to produce substances such as bacteriocins, enzymes, lactic acid, and fatty acids, prevent the colonization of certain commensal microorganisms [26]. This may account for the lower diversity observed in formula-fed piglets. Bacterial diversity can impact the host immune system, for example, in the primate model [27], dam-reared rhesus macaques had a distinct colonic microbiota compared to those bottle-fed, and disparate immune systems were observed even after weaning from neonatal diet, suggesting that initial microbial colonization impacts immune system.

Previously in small intestine we observed a significant morphological changes in formula-fed piglets along with an increased expression of cytokines and decreased expression of anti-inflammatory molecule IL-10 (mRNA and protein). Interestingly no major tissue morphological changes or membrane protein expression were noted in the colon with formula feeding. Thus, it is possible that tissue defense mechanisms protect the colon. For instance, alkaline phosphatase-detoxifes LPS, ameliorates intestinal inflammation, and regulates gut microbial communities and their translocation across the gut barrier [28]. Interestingly, alkaline phosphatase activity is increased approximately threefold in distal colon contents of milk-fed piglets relative to the sow-fed group. Furthermore, dairy products contain a high concentration of alkaline phosphatase [29] that may account for the increased alkaline phosphatase activity observed in the colon contents. The presence of more HSP-27 in the soy fed piglets may be an indication of another defense mechanism in this group. In addition, increased BMP4, (known to cause intestine barrier dysfunction), and CCL25, (a chemokine known to regulate the trafficking of gut-specific memory/effector T cells into gut mucosa) expression in soy- and milk-fed piglets was noted. Of relevance to these results are findings that an exclusively breastfed infants (at 3 months of age), but not exclusively formula-fed infants, showed down-regulation of genes such as KLRF1, BPL1, ALOX5, IL-1α, and AOC3 that prime mucosal inflammatory responses [30]. Overall, data suggests that formula diet impacts immune response relative to breastfeeding.

The microbiota fuctional prediction analyses showed several pathways that differed significantly between the diet groups. We observed more genera in milk-fed piglets (3 vs 1) correlating with apoptosis relative to the sow-fed group, suggesting the colon microbiota may increase the exfoliation of epithelial cells from gut mucosa and possibly reduce EC cell number as we observed here. In the sow-fed group, only *Butyricimonas* was positively correlated with bacterial invasion of epithelial cells, while in soy and milk groups four genera were positvely correlated, suggesting a higher chance of epithelial cell invasion in formula groups. It is well accepted that gut microbiota-programming of host epithelial cell transcription is region-specific [31]. Thus, the specific set of microbiota present in the colon could have diverse physiological effects on the host. However, it should be noted that the pathways are based on prediction analyses and future studies will be needed to confirm the impact on the specific pathways.

Based on microbiota prediction analyses, we focused on tryptophan metabolism and interestingly, several genera were correlated to tryptophan metabolism. Consistent with our data Poroyko et al. reported that formula group cecal microbiota was enriched with sequences for aromatic amino acid degradation. Thus, to understand tryptophan fate in the distal colon, we looked at the serotonin pathway and observed significantly lower levels of serotonin in colon tissue of formula-fed piglets. However, no differences were observed in serum serotonin levels, suggesting that the small intestine (and other tissue) contributed to the serum serotonin levels. Furthermore, serotonin precursors-tryptophan and 5HTP were unaffected in distal colon contents, but increased in serum and urine of soy and milk groups. It is possible that 5HTP from the colon is transported into serum more readily in soy and milk groups and excreted into urine and thus lower levels of serotonin were observed in these groups. Moreover, 5HIAA, a catabolic product of serotonin level was not altered in distal colon contents and urine and undetectable in serum, ruling out the possibility of low serotonin due to increased catabolism of serotonin into 5HIAA in formula-fed piglets. We also noted significantly increased levels of tryptamine in formula group relative to sow in colon contents, but not in tissue. However, to understand if proximal gut regions contribute to tryptamine levels present in the colon, tryptamine was measured from duodenum contents. These analyses showed lower level of tryptamine in duodenum contents in all groups. In addition, in serum and urine tryptamine levels did not differ among the diet groups. Moreover, the amount of tryptamine observed in the diet or distal colon tissues is minimal in comparison to distal colon contents, suggesting that diet or tissue possibly do not contribute to high levels of tryptamine observed and highlighting the fact that tryptamine is possibly produced by distal colon microbiota. Furthermore, *Clostridium, Ruminococcus, Bilophila, Butyrricimonas, and Blautia* genera are positively correlated with tryptophan metabolism. Interestingly, *Clostridium sporogenes and Ruminococcus gnavus* are known to consume tryptophan to produce tryptamine [32]. However, mammals convert tryptophan to tryptamine [33] and tryptamine produced by host cells could be readily transported to the blood and be released into the lumen. Thus, levels of tryptamine observed are possibly due to microbiota or host. Future studies are required to determine if tryptamine is derived from host or bacterial species under these conditions.

It is known that serotonin receptors contribute to the immune system regulation [34]. Interestingly, the serotonergic receptor-mediated signaling pathway is not only activated by endogenous agonist serotonin, but also by an exogenous tryptamine. The relative affinity of serotonin and tryptamine for serotonin receptors could cause imbalance of local and systemic serotoninergic system and thereby affect the immune system and other physiological functions. Most recently, using the Ussing-chamber model it was reported that tryptamine causes ion release in intestinal epithelial cells, suggesting tryptamine might affect the food particles transit or gastric motility [32]. In addition, an early study conducted in 1950 showed that tryptamine injection into skin flap or gastronemius muscle of cat resulted in histamine release [35] and it is well known that increased histamine levels are reported in allergic conditions [36]. Previous reports have indicated that tryptamine might play a role in mood and appetite [37, 38] as it can cross the blood brain barrier unlike serotonin (5-HT). Interestingly tryptamine could also promote 5-HT release from enterochromaffin cells, and possibly have impact locally on the GI tract [37]. However, future studies are needed to determine the programming effect of the microbiota and the tryptophan metabolites observed during neonatal period and how that impacts host immune system and allergies. In addition, how tryptamine may differ from serotonin in the activation of cellular signaling pathways and the kyneurine pathway (another tryptophan pathway) and how this activation impacts in these piglets needs further investigation.

## Conclusions

In summary, we studied the microbiota composition in distal colon and focused on aromatic amino acid metabolism (tryptophan). We demonstrated that relative to sow feeding, formula feeding: (1) alter colon microbiota richness and diversity; (2) affect colon cytokine response (3) reduce host serotonin levels; and (4) increase tryptamine production. In conclusion, these data suggest that the formula-associated colon microbiota possibly impacts sertonin biosynthesis and favors the bacterially-derived typtamine production, and thereby altering the colon’s immune response. If recapitulates in human infants, these early changes in GI and immune system development due to formula feeding could have long-term health consequences.

### Limitations

Many factors other than diet can contribute to the development of gut microbiota composition such as housing environment, maternal proximity, pens, and mother’s diet. In this study the sow-fed piglets were housed at the farm until day 21 and formula-fed were brought to the animal facility on day 2. There is a need to use more controlled environment to understand microbiota changes due to the dietary differences, which could be achieved by using human breast milk-fed piglet model. Thus, future studies will focus on human breast milk-fed piglet model.

## Additional file

Additional file 1: Formula diet alters tryptophan metabolism. (ZIP 709 kb)

## Abbreviations

5-HIA: 5-hydroxyindole aldehyde
5-HIAA: 5-Hydroxyindole acetic acid
5-HT: 5-Hydroxy tryptamine/serotonin
5-HTP: 5-Hydroxytryptophan
AADC: Aromatic acid decarboxylase
ADH: Aldehyde dehydrogenase
ALOX5: Arachidonate 5-lipoxygenase
AOC3: Amine oxidase copper containing 3
BMP4: Bone morphogenetic protein 4
BPL1: Biotin protein ligase 1
CCL25: C-C motif chemokine ligand 25
CgA: Chromogrannin A
DC: Distal colon
DD: Duodenum
EC: Enterochormaffin cells
GI: Gastro-intestine
HSP-27: Heat shock protein 27
IACUC: Institutional Animal Care and Use Committee
IL-1α: Interleukin-1α
KLRF1: Killer cell lectin-like recetor F1
LPS: Lipopolysaccharide
MAO: Monoamine oxidase
TPH: Tryptophan hydroxylase
