# Supplementary material for: Formula diet driven microbiota shifts tryptophan metabolism from serotonin to tryptamine in neonatal porcine colon﻿
Source: Microbiome. 2017 Jul 14;5:77. doi: 10.1186/s40168-017-0297-z (PMC5513086; doi:10.1186/s40168-017-0297-z)
Supplement: Additional file 1: — Formula diet alters tryptophan metabolism. (ZIP 709 kb) [file 40168_2017_297_MOESM1_ESM.zip › Supplemental file revised final.docx]

**SUPPLEMENTAL RESULTS**

***Microbiota***

At class level, *Bacteroidia* was the most abundant in sow (52.9%), milk formula (66.1%) and soy formula (65.1%). *Clostridia* was the next abundant microbes. However, the relative abundance of *Clostridia* did not change among the groups (19.3%-Sow;16.9%-Milk formula; 20.8%-soy formula). *Fusobacteria* was 7.3% abundant in sow fed piglets but decreased in milk formula 0%, and soy formula 0.3%). *Betaproteobacteria* was also not affected by formula diet (6.4% sow; 5.9% soy; 6% milk). *Gammaproteobacteria* (3.3% milk formal, 1.8% soy formula vs 0.35% sow) and *Verrucomicrobiae* (2.3% milk formula, 0.93% soy formula vs 0.13% soy) significantly (log2 fold 2 to 6 fold, p<0.05) increased in milk or soy formula fed piglets vs sow piglets. However low abundant bacteria (<3%) *Spirochaetes, [Lentisphaeria], Verruco-5, 4C0d-2, Planctomycetia, RF3* and *Alphaproteobacteria* reduced significantly (log2fold 2 to12 vs sow, p<0.05) in both formula fed group. Interestingly, *Coriobacteriia* (an *actinobacteria*) significantly decreased (log2 fold 2.23) only in milk formula fed group. Another *Actinobacteria* and *Synergistia* (a Synergistetes) suppressed in soy fed piglets relative to sow fed piglets. *Epsilonproteobacteria* (a protobacteria) decreased in milk fed group **(Supplemental Figure S1A).**

At order level, *Bacteroidales* (52.9% sow, 66.1% milk formula, 65.1% soy formula)*, Clostridiales* (19.3% sow, 16.9% milk formula, 20.8% soy formula)*, Burkholderiales* (6.4% sow, 6% milk formula, 5.9% soy formula)*, Lactobacillales* (2.9% sow, 2.1% milk formula, 1.4% soy formula) *Desulfovibrionales* (1.51%, 2.7% and 1.75% respectively) were highly abundant and unaltered by formula feeding. *Enterobacteriales* was significantly higher in milk formula fed group (3.3% vs 0.058%, p<0.05; log2 fold 5.76) and soy formula fed group (1.5% vs 0.058%, p<0.05; log2 fold 4.9). *Verrucomicrobiales* increased in milk fed (2.3% vs 0.1%, p<0.05, log2 fold 5.43) and soy fed piglets (0.9% vs 0.1%, log2 fold 1.03, p<0.05). The low abundant microbes such as *WCHB1.41, Victivallales, Campylobacterales, Pirellulales, GMD14H09, Spirochaetales, Pasteurellales, ML615J.28, YS2, Z20* were either absent or almost negligible in formula group in comparison to sow group (0.1%, 0.69% vs 6.9%). The analysis of microbiota data by negative binomial wald test exhibited 2 to 12 log 2 fold decrease (p<0.05) *Victivallales, WCHB1-41, YS2, Spirochaetales, Z20, GMD14H09, Pirellulales, Pasteurellales* in both formula group. When we compared the microbiota of milk-fed group with soy fed group, Interestingly, *Campylobacterales* was increased (log2 fold 9.95) and *Pseudomonadales* was log2 fold 4.45 **(Supplemental Figure S1B).**

At family level, *Prevotellaceae* (sow 19.2%; milk formula 16.6%; soy formula 24.8%), *Bacteroidaceae* (sow 10.9%; milk formula 21.1%; soy formula 15.1%), *Ruminococcaceae* (sow 10.3%; milk formula 8.3%; soy formula 12.8%) were highly abundant and did not change much with formula diets. *Porphyromonadaceae* was significantly higher in both milk formula and soy formula-fed piglets (10.1% vs 1.4%; 11.1% vs 1.4%, p<0.05, log2 fold ~ 2.5). *Rikenellaceae* was 8.9% in milk-fed group which was 3.9 log2 fold higher (p<0.05) as compared to sow fed group (0.5%). *Enterobacteriaceae* increased in milk formula (3.4% vs 0.067%, p<0.05; log2 fold 5.18)) and soy formula (1.5% vs 0.067%, p<0.05; log2 fold 4.66)) fed piglets. In compassion to sow fed group, S24.7 reduced significantly in both milk and soy formula group (0.0% vs 9.2%, log2 fold 8.59; 1.1% vs 9.2%, log2 fold 2.26; p<0.05). *[Paraprevotellaceae]* also reduced only in milk formula group (0% vs 5.2%; log2 fold 14.58; p<0.05). *RFP12* (1.7%; p<0.05), *Pirellulaceae* (0.54%; p<0.05), *Spirochaetaceae* (0.47% p<0.05), *R4.45B* (0.12%; p<0.05) were present only in sow fed piglets and an almost negligible or absent in formula-fed piglets. The analysis of microbiota data by negative binomial wald demonstrated that *Victivallaceae, RFP12 Fusobacteriaceae Campylobacteraceae p-2534-18B5* were reduced (log 2 fold 6 to 12) in both milk and soy formula fed group. *Campylobacteraceae* (log2 fold 10.59), *Peptococcaceae* (log2 fold 5.23) *Pasteurellaceae* (log 2 fold 2.13) were reduced significantly (p<0.05) only in milk formula group versus sow group. In contrast *Streptococcaceae, [Odoribacteraceae], Moraxellaceae, Verrucomicrobiacea*e and *Enterococcaceae* were increased significantly (log 2 fold 2 to 5; p<0.05) in milk formula-fed piglets versus sow fed piglets. On the other side, in soy-fed group *Streptococcaceae, Clostridiaceae, Veillonellaceae and Verrucomicrobiaceae* were increased around log2 fold 2 to 3.5 (p<0.05). Interestingly, *Synergistaceae, Christensenellaceae* and *Micrococcaceae* were reduced only in soy formula group (log2 fold 2 to 4; p<0.05). On comparison of soy group versus milk group *[Paraprevotellaceae], S24-7,Campylobacteraceae* and *Helicobacteraceae* were increased, while *Christensenellaceae* and *Moraxellaceae* were decreased soy formula versus milk formula piglets **(Supplemental Figure S1C).**

**SUPPLEMENTAL FIGURE LEGENDS**

**Figure S1: Microbial diversity and relative abundance in piglets fed with sow, soy, and milk based diets.** Stacked bar charts represent relative abundance at **(A)** Class, **(B)** Order and **(C)** Family level (n = 12/group).

**Figure S2: Histomorphometric and membrane protein analyses in piglets fed with sow, soy and milk diets. (A)** H&E stained slides exhibits the morphological features of distal colon of sow, soy and milk fed piglets. The bar graphs demonstrate (**B)** distal colon length **(C)** Ratio of crypt depth to thickness **(D)** ratio of crypt count to length **(E)** crypt circumference **(F)** relative protein expression of HSP27 **(G)** relative protein expression of β-catenin (**H)** Images demonstrates distal colon HSP-27 immunostaining in sow, soy, and milk fed piglets. Data were analyzed by one way ANOVA followed by post hoc test (n=12/group, * p < 0.05, ** p<0.01 for formula diet in comparison to control sow fed piglets).

**Figure S3: Predictive function profiling of 16s RNA OTU data.** PiCRUST was used to generate a profile of putative functions (via metagenome prediction) from the 16srRNA OTU data. OTUs were picked from a demultiplexed fasta file containing the sequences for all distal colon contents samples using the closed-reference protocol where we searched sequences against the GG reference OTUs at 97% percent identity. These OTUs were normalized by the predicted 16S copy number. BIOM table containing the predicted metagenome for each sample was attained. We used STAMP software to determine statistical significance of functional metagenomics prediction data. The significant differences in functional category relative abundances among diet groups at level-3 profile were determined by ANOVA followed by Tukey-Kramer post-hoc test with effect size Eta-squared to test (n=12/group).

**Figure S4: Tryptophan and its metabolites in piglets fed with sow, soy and milk diets.** Data from detectable peaks were only included. (**A)** Bar graph demonstrates serotonin level in duodenum contents (n =11-12/group) and urine (n=7-8/group). **(B)** Distal colon contents 5-HTP (n_sow_=4, n_soy_=7, n_milk_=11), 5-HIAA (n_sow_=8, n_soy_=9, n_milk_=5), and tryptophan (n =11-12/group) levels. **(C)** Serum 5-HTP (n=12/group), 5-HIAA (n=12/group, not detectable), and tryptophan (n=12/group) levels. **(D)** Urine 5-HTP (n_sow_= not detectable, n_soy_=12, n_milk_=12), 5-HIAA (n=9-12/group), and tryptophan (n =10-12/group) levels. Tryptamine level was displayed in bar graph in **(E)** duodenum (DD) contents (n = 9-11/group), serum (n=12/group), and urine (n = 10-12/group). Indole acetic acid levels were displayed in bar graph for **(F)** serum (n=12/group) and urine (n = 10-12/group). The data were analyzed by one way ANOVA followed by post hoc test (* p < 0.05, ** p<0.01, *** p<0.002 for formula diet in comparison to control sow fed piglets).

**SUPPLEMENTAL TABLES**

| **Supplementary Table 1:** Sample richness and diversity of microbiota at all taxonomical level. | | | | |
| --- | --- | --- | --- | --- |
| Index | Sow | Milk | Soy | *P* |
| ***Phylum*** | | | | |
| Chao1 | 7.6 ± 0.4 | 6.8 ± 0.22 | 5.9 ± 0.36 | 0.00407 |
| Shannon | 1.1 ± 0.056 | 0.91 ± 0.024 | 0.9 ± 0.043 | 0.02276 |
| Simpson | 0.58 ± 0.024 | 0.51 ± 0.016 | 0.51 ± 0.02 | 0.05188 |
| InvSimpson | 2.4 ± 0.14 | 2.1 ± 0.068 | 2.1 ± 0.099 | 0.03071 |
| Fisher | 0.76 ± 0.046 | 0.67 ± 0.025 | 0.57 ± 0.037 | 0.00270 |
| ***Class*** | | | | |
| Chao1 | 12 ± 0.58 | 11 ± 0.3 | 11 ± 0.37 | 0.04826 |
| Shannon | 1.2 ± 0.054 | 1.1 ± 0.036 | 1.1 ± 0.05 | 0.36882 |
| Simpson | 0.59 ± 0.026 | 0.53 ± 0.019 | 0.53 ± 0.021 | 0.12412 |
| InvSimpson | 2.5 ± 0.16 | 2.2 ± 0.09 | 2.2 ± 0.11 | 0.07363 |
| Fisher | 1.3 ± 0.06 | 1.1 ± 0.03 | 1.1 ± 0.035 | 0.01798 |
| ***Order*** | | | | |
| Chao1 | 12 ± 0.4 | 11 ± 0.3 | 11 ± 0.35 | 0.33897 |
| Shannon | 1.1 ± 0.052 | 1.1 ± 0.034 | 1.1 ± 0.05 | 0.60955 |
| Simpson | 0.58 ± 0.026 | 0.53 ± 0.018 | 0.53 ± 0.021 | 0.17292 |
| InvSimpson | 2.5 ± 0.16 | 2.2 ± 0.082 | 2.2 ± 0.11 | 0.10467 |
| Fisher | 1.3 ± 0.052 | 1.2 ± 0.033 | 1.2 ± 0.034 | 0.21525 |
| ***Family*** | | | | |
| Chao1 | 25 ± 0.46 | 23 ± 0.74 | 23 ± 0.36 | 0.01445 |
| Shannon | 2.1 ± 0.099 | 2.2 ± 0.045 | 2.2 ± 0.034 | 0.62448 |
| Simpson | 0.82 ± 0.02 | 0.83 ± 0.01 | 0.83 ± 0.011 | 0.75694 |
| InvSimpson | 6.2 ± 0.55 | 6.3 ± 0.42 | 6.2 ± 0.31 | 0.98981 |
| Fisher | 2.9 ± 0.084 | 2.6 ± 0.047 | 2.6 ± 0.048 | 0.00102 |
| ***Genus*** | | | | |
| Chao1 | 42 ± 1 | 37 ± 0.71 | 39 ± 0.7 | 0.00006 |
| Shannon | 2.2 ± 0.12 | 2.3 ± 0.04 | 2.3 ± 0.04 | 0.66395 |
| Simpson | 0.83 ± 0.021 | 0.84 ± 0.0086 | 0.84 ± 0.011 | 0.78741 |
| InvSimpson | 6.7 ± 0.69 | 6.6 ± 0.42 | 6.6 ± 0.38 | 0.97294 |
| Fisher | 5.1 ± 0.2 | 4.3 ± 0.075 | 4.6 ± 0.11 | 0.00264 |

| **Supplementary Table 2:** Primers sequences used to measure gene expression. | |
| --- | --- |
| Gene | Primer sequence (5’- 3’) |
| FASLG-F | GCCAGCCAAAGGCATACAGA |
| FASLG-R | CCTGTTAAGTGGGCCACTTTTC |
| IL10-F | GAGGATATCAAGGAGCACGTGAAC |
| IL10-R | CTCTTGTTTTCACAGGGCAGAA |
| IL27-F | AACGACTCTGCTTCCTCTCCAT |
| IL27-R | CAGCTGCATCCTCTCTGAACTG |
| LOC100152038-F | CTGCACAGACTGACTGCTTTACAG |
| LOC100152038-R | TGGGACATGCAATGGATGTTAC |
| LOC100736831-F | CGAGCCAGGGATGAAAGGT |
| LOC100736831-R | TGCCAAGTGCAAGAACACATAGT |
| TNF-F | AAGGACTCAGATCATCGTCTCAAA |
| TNF-R | GGCATACCCACTCTGCCATT |
| IL6-F | CTGCTTCTGGTGATGGCTACTG |
| IL6-R | TCCGGAGAGGTGAAGAGCATT |
| CCL11-F | CAGCTTCTGTCGCCACCAT |
| CCL11-R | GGGACATTTGTTGGCAGTGACT |
| BMP4-F | ATGATTCCTGGTAACCGAATGC |
| BMP4-R | TTTTTCTTCCCCGTCTCAGGTA |
| CCL21-F | CCATCCCAGCTATCCTGTTCTC |
| CCL21-R | GTCCAGATGGCGCATCAGTT |
| CSF3-F | CTGCTGCTCTGGCACATTG |
| CSF3-R | TTCCTCACTTGCTCTAAGCACTT |
| TNFSF10-F | GGCATTCCTCACCTAGAAAGAGAA |
| TNFSF10-R | TTGGAGCTTAGAGATGGAAATGTG |
| CCL25-F | GATCCTGGACAATCGGAATAAGA |
| CCL25-R | TTCCAGAGCTCAACTTCCTCACT |
| CD40LG-F | ATCCTCAAATTGCGGCACAT |
| CD40LG-R | CAAGTTGGTGCTGAGGGTGTAG |
| VEGFA-F | GACGTCTACCAGCGCAGCTACT |
| VEGFA-R | ACACAGGACGGCTTGAAGATGT |
| CXCL11-F | GGCTGTCATATTTTGTGCTACA |
| CXCL11-R | CTGCCACTTTCACTGCTTTTAC |
| ACTB-F | TCTTCCAGCCCTCCTTCTTG |
| ACTB-R | GCGTAGAGGTCCTTCCTGATGT |
| Tph1-F | GTC CTG TGG CTG GTT ACT TAT C |
| Tph1-R | CGA ACC GTG TCT CAC ATA CTG |
| NKX2-F | TCT CTC TCT CTC TCT CTC TTT CC |
| NKX2-R | CCG AGC GAT CAG TCC ATA TAA |
| Lmx1a-F | CTG TCC TTG GAG CAG GTA ATA G |
| Lmx1a-R | CTG TCC TTG GAG CAG GTA ATA G |
| VMAT_1_-F | CAG GGA GCT AAG GTT CCA ATT A |
| VMAT_1_-R | CTC CTC TCT GAA AGT GGA TGT G |
