# Supplementary figures and images for: Formula diet driven microbiota shifts tryptophan metabolism from serotonin to tryptamine in neonatal porcine colon﻿
Source: Microbiome. 2017 Jul 14;5:77. doi: 10.1186/s40168-017-0297-z (PMC5513086; doi:10.1186/s40168-017-0297-z)

**A**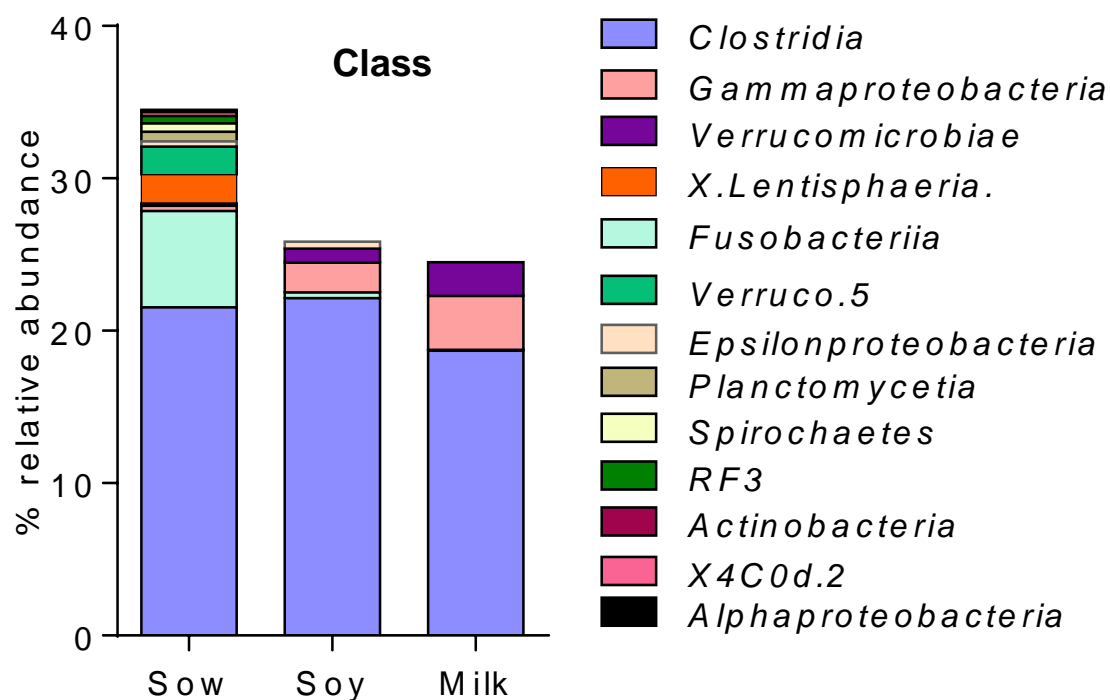**B**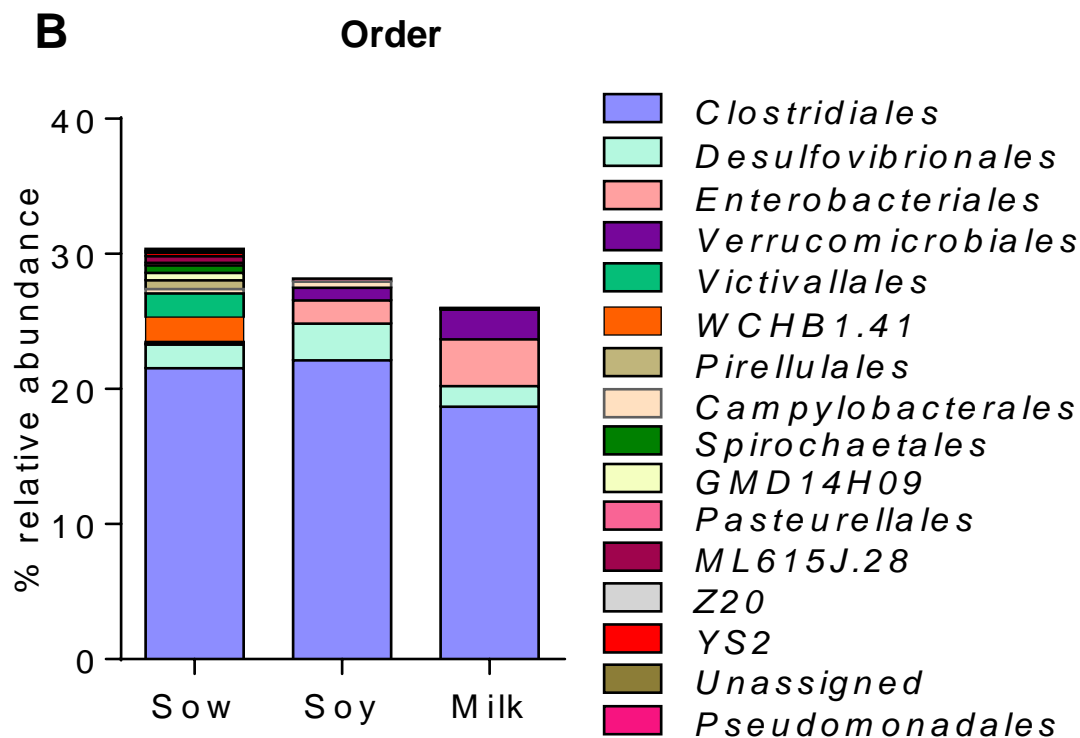**C**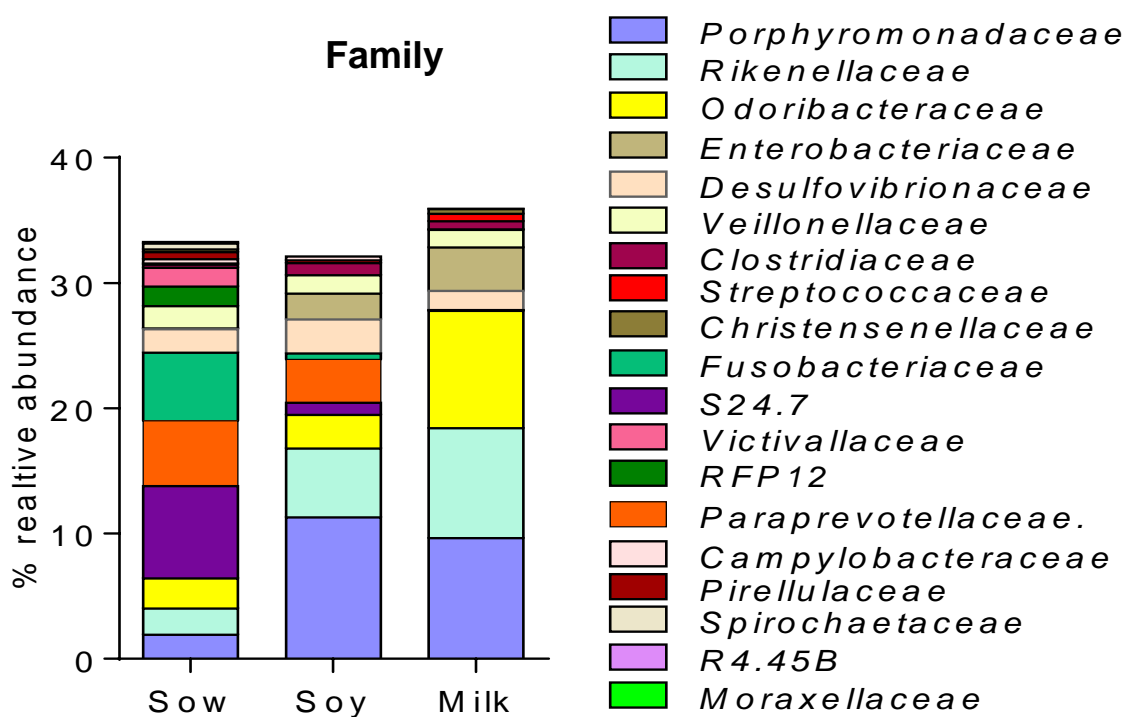

Supplement: Additional file 1: — Formula diet alters tryptophan metabolism. (ZIP 709 kb) [file 40168_2017_297_MOESM1_ESM.zip › Suplementary Figure S1.pdf]

**A**

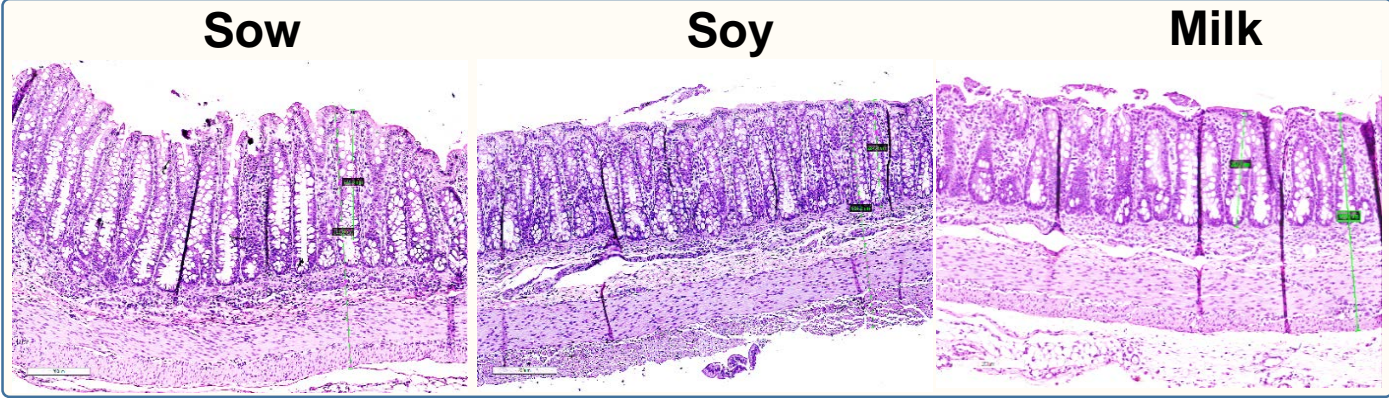

**B**

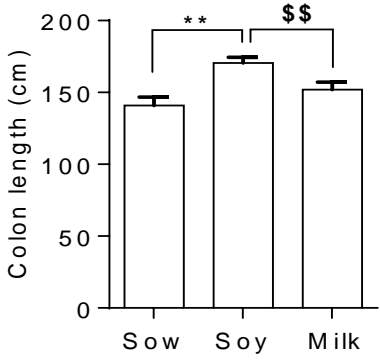

**C**

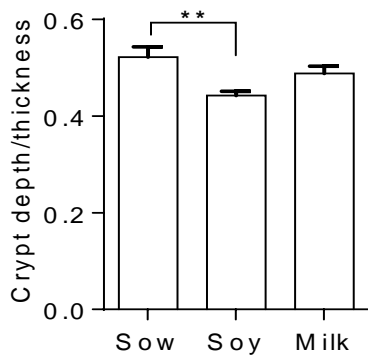

**D**

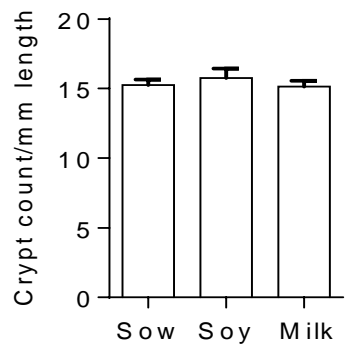

**E**

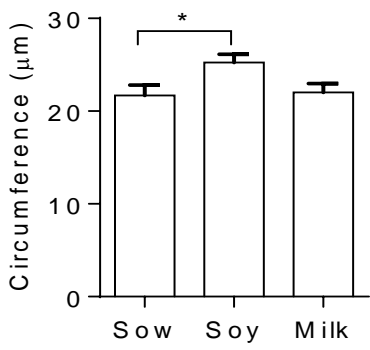

**F**

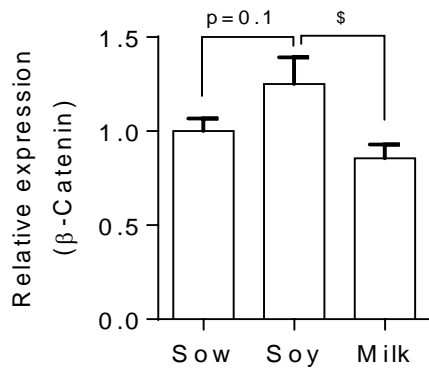

**G**

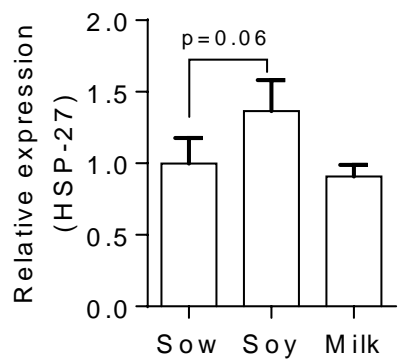

**H**

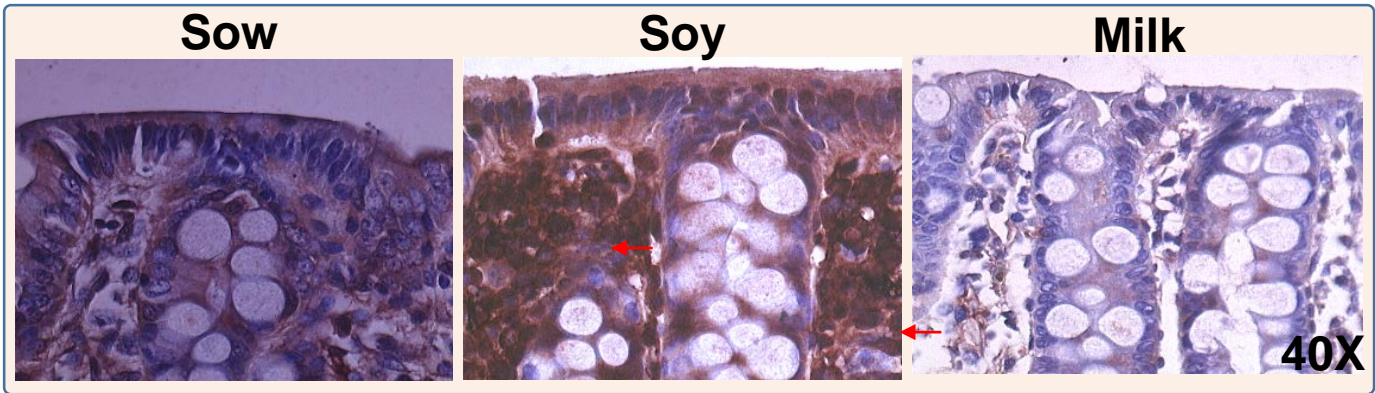

Supplement: Additional file 1: — Formula diet alters tryptophan metabolism. (ZIP 709 kb) [file 40168_2017_297_MOESM1_ESM.zip › Suplementary Figure S2.pdf]

A

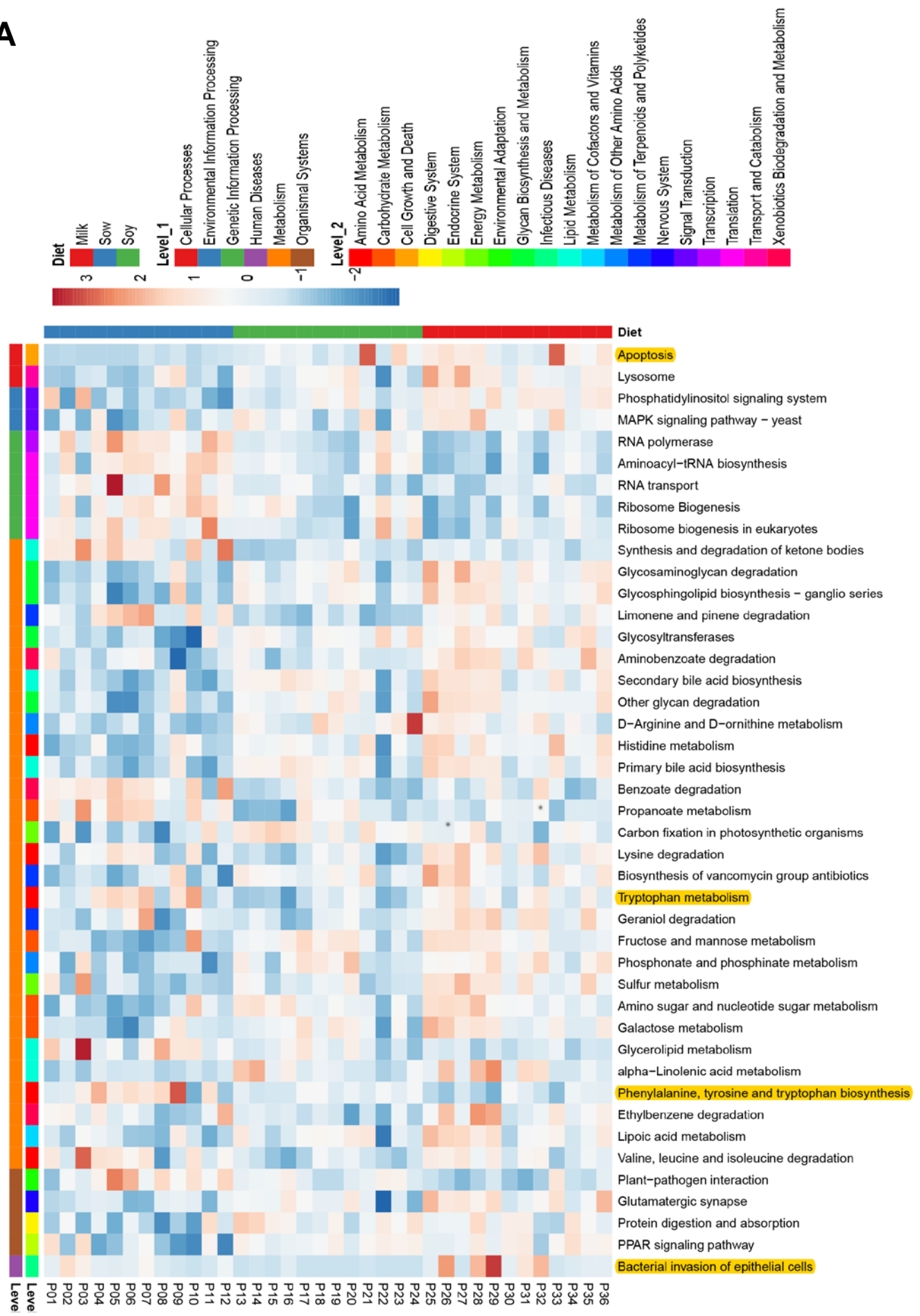

B

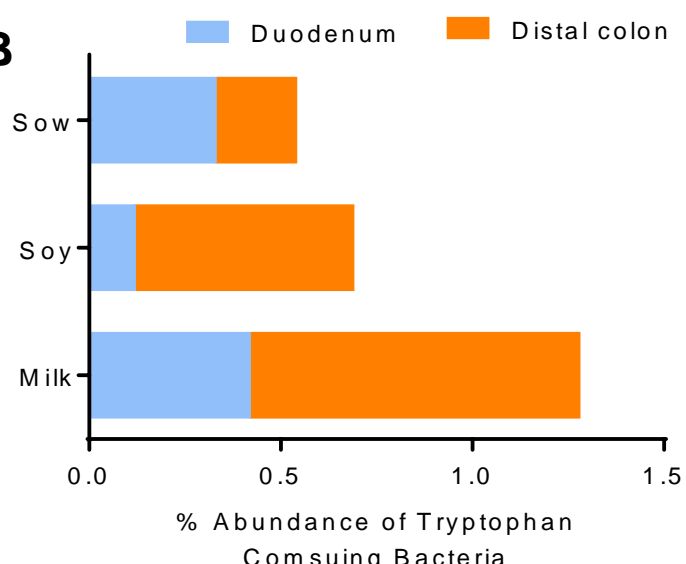

Supplement: Additional file 1: — Formula diet alters tryptophan metabolism. (ZIP 709 kb) [file 40168_2017_297_MOESM1_ESM.zip › Suplementary Figure S3.pdf]

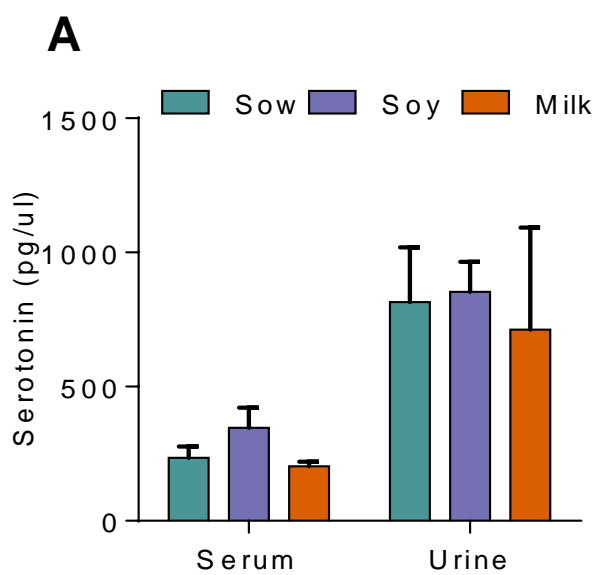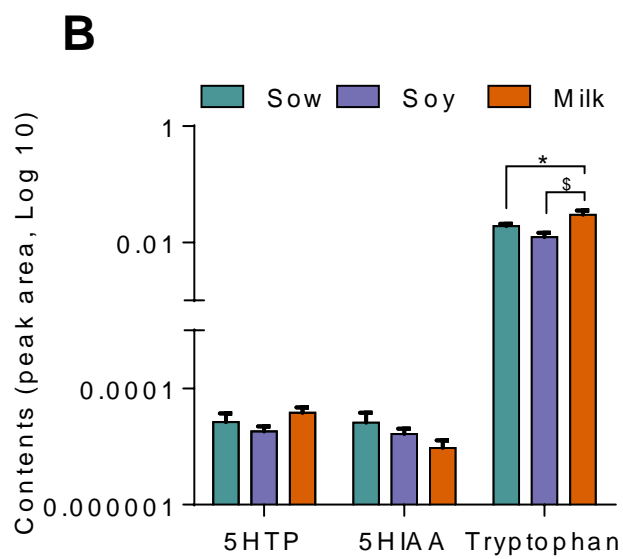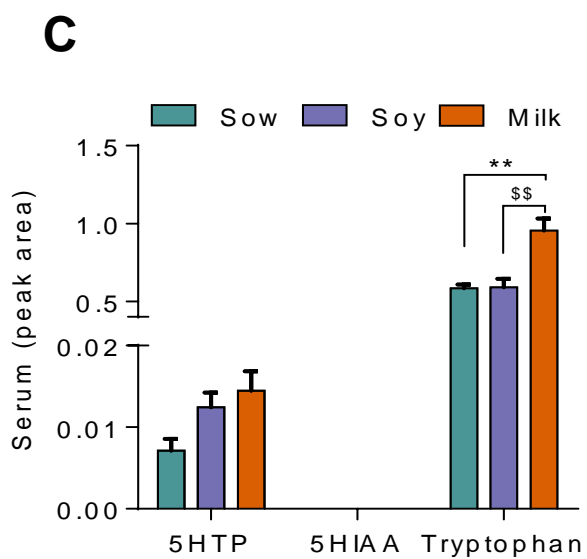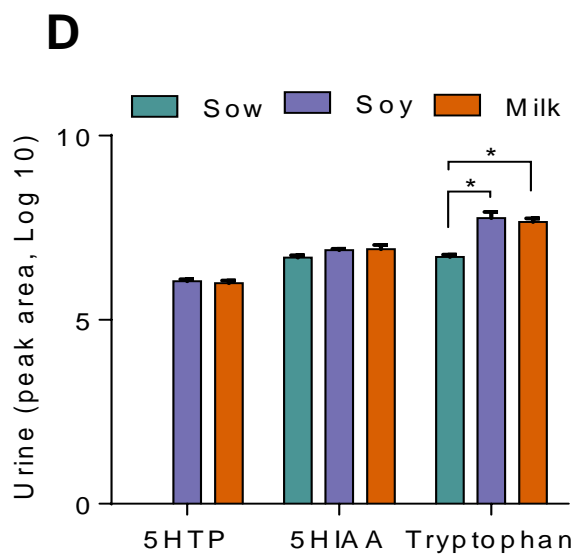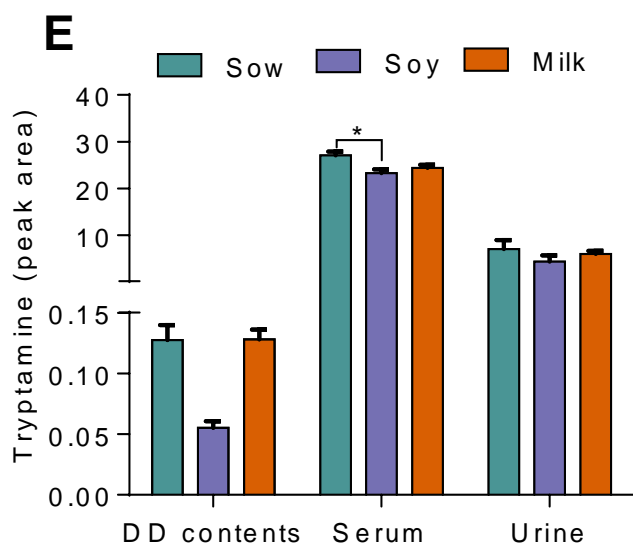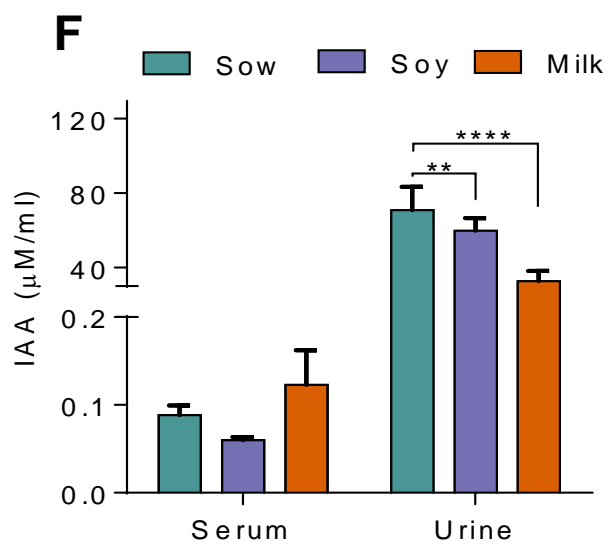

Supplement: Additional file 1: — Formula diet alters tryptophan metabolism. (ZIP 709 kb) [file 40168_2017_297_MOESM1_ESM.zip › Suplementary Figure S4.pdf]
